# Identification and Validation of Reference Genes for Quantitative Gene Expression Analysis in *Ophraella communa*

**DOI:** 10.3389/fphys.2020.00355

**Published:** 2020-05-07

**Authors:** Yan Zhang, Jiqiang Chen, Guangmei Chen, Chao Ma, Hongsong Chen, Xuyuan Gao, Zhenqi Tian, Shaowei Cui, Zhenya Tian, Jianying Guo, Fanghao Wan, Zhongshi Zhou

**Affiliations:** ^1^State Key Laboratory for Biology of Plant Diseases and Insect Pests, Institute of Plant Protection, Chinese Academy of Agricultural Sciences, Beijing, China; ^2^Guangxi Key Laboratory of Biology for Crop Diseases and Insect Pests, Institute of Plant Protection, Guangxi Academy of Agricultural Sciences, Nanning, China

**Keywords:** *Ophraella communa*, RT-qPCR, reference gene, normalization, gene expression

## Abstract

*Ophraella communa* is an effective bio-control agent of the invasive common weed. By now, the reference genes in *O. communa* have not yet been screened and validated. The aim of this study was to screen for the most stable reference genes in different backgrounds, such as different developmental stages, sexes, tissues, and male reproductive system with different body sizes. We selected 12 common housekeeping genes involved in different biological processes, including *GAPDH*, *ACT1*, *ACT2*, *ARF1*, *ARF4*, *SDH*, *βTUBC*, *RPL4*, *RPL19*, *RPS18*, *EF1*α, and *COX* as the candidate reference genes. To analyze the stability of the candidate reference genes, we first used three dedicated algorithms, GeNorm, NormFinder, and BestKeeper, and further comprehensive ranking was provided by ReFinder. The results showed that *RPL19* and *RPL4* exhibited the least variation in different developmental stages/sexes and in male reproductive systems with different body sizes. *COX* proved to be most suitable for normalizing the gene expression levels in different tissues, and coincidentally, *RPL19* was also found to be second in terms of stability in this study. To the best of our knowledge, this is the first study to identify suitable reference genes for analyzing gene expression in *O. communa*; thus, this study would lay the foundation for future research on the molecular physiology and biochemistry of *O. communa* and other insects.

## Introduction

*Ophraella communa* LeSage (Coleoptera: Chrysomelidae) originated in North America (LeSage, 2012). It is a biological control agent of the invasive weed *Ambrosia artemisiifolia* L. (common ragweed) (Yamazaki et al., 2000; Zhou et al., 2010a, 2014; Gerber et al., 2011; Guo et al., 2011). For a biological control agent, reproductive regulation is a very important issue in order to facilitate its mass rearing. Gratifyingly, we have found that the *O. communa* males with a large body size can facilitate a female’s fecundity (unpublished data). Therefore, how to regulate the reproductive potential of the male beetle will be focused on in a further study. It is necessary to assess gene function in the beetle; quantification of gene expression is required as well.

In recent years, the ecology and physiology of *O. communa* has been studied extensively (Zhou et al., 2010a,b; Clark et al., 2014; Kim and Lee, 2018; Zhao et al., 2018). However, the molecular mechanisms of its biology remain largely unknown (Ma et al., 2019a,b). With the aim of investigating the genetic basis of its biology and physiology, the male transcriptome and proteome of *O. communa* have been sequenced (unpublished data). Remarkably, we found that the male’s body size is an important determinant in the evolutionary process and in the speciation of *O. communa*, as it affects female mate choice. Then, the large males could further improve the population fitness of their offspring (unpublished data). Currently, the mechanisms for higher fitness in large males are also being studied, so it is necessary to assess gene function in *O. communa*; quantification of gene expression is required as well.

Reverse transcriptase-quantitative polymerase chain reaction (RT-qPCR), also referred to as qPCR, owing to its excellent repeatability, high sensitivity, accurate quantification, wide applicability, and easy operation, is the most common and sensitive technique for the detection and quantification of gene expression in different experimental samples, such as developmental stages, tissues, and other processing conditions (Bustin, 2002). It is especially useful to detect low-abundance mRNAs in limited samples (Bustin et al., 2005; Valasek and Repa, 2005; Chen et al., 2011; Hellemans and Vandesompele, 2014). However, the results of qPCR are often limited by factors such as the amount of the initial sample, cDNA synthesis efficiency, and primer amplification efficiency, which may result in inaccurate qPCR results (Bustin et al., 2005). Therefore, this approach requires normalization.

To date, internal control genes, also known as housekeeping genes or reference genes, are genes whose expression levels are stable regardless of the cell type and the experimental conditions. It is the most popular tool to normalize variability (Thellin et al., 1999; Vandesompele et al., 2002; Andersen et al., 2004; Huggett et al., 2005; Hellemans and Vandesompele, 2014). Many genes such as *Actin*, *GAPDH*, and *rRNAs* (18s or 28s) that are involved in fundamental biological processes are frequently used as reference genes (Keertan et al., 2004; de Kok et al., 2005). Nevertheless, it has become clear that no single reference gene retains a constant expression in all tissues, developmental stages, or experimental conditions (Silver et al., 2006; Lourenço et al., 2008; Lu et al., 2013, 2018a; Rodrigues et al., 2014; Tan et al., 2015; Dai et al., 2017; Luo et al., 2018). Therefore, the traditional genes are not applicable to all species and conditions (Shu et al., 2018; Zhou et al., 2018; Qu et al., 2019). Blindly choosing “classical” reference genes such as β-actin, glyceraldehyde-3-phosphate dehydrogenase (GAPDH), or ribosomal 18S (18S) is unreliable and inaccurate. The reliability of qPCR largely depends on the stability of the reference genes. Previous studies have shown that it is crucial to select and validate the reference gene for data quantification (Lu et al., 2018b; Shakeel et al., 2018; Shu et al., 2018). Hence, it is extremely important to verify the stability of expression of the intended control gene under different experimental conditions. To our knowledge, research on the reference gene of *O. communa* is absent, which makes the study of the mechanisms of its physiological and biochemical behavior difficult.

In this study, we aim to identify the solidly expressed reference genes for RT-qPCR investigation under different conditions including developmental stages, sexes, tissues, and male reproductive system with different body sizes of *O. communa*. Based on prior experimental reports regarding reference genes in coleoptera and other insects, 12 candidate reference genes including glyceraldehyde-3-phosphate dehydrogenase (*GAPDH*), actin 1 (*ACT1*), actin 2 (*ACT2*), succinate dehydrogenase (*SDH*), ADP-ribosylation factor 1 (*ARF1*), ADP-ribosylation factor 4 (*ARF4*), cytochrome oxidase (*COX*), β-tubulin C (β*TUBC*), elongation factor-1α (*EF1*α), ribosomal protein L4 (*RPL4*), ribosomal protein L19 (*RPL19*), and ribosomal protein S18 (*RPS18*), which are involved in various biological processes, were selected from the male transcriptome of *O. communa*. The results of the qPCR were analyzed using three statistical algorithms, GeNorm (Vandesompele et al., 2002), NormFinder (Andersen et al., 2004), and BestKeeper (Pfaffl et al., 2004). Finally, a comprehensive ranking of the stability of these candidates was given by ReFinder (Yang et al., 2015a). In addition, the expression profile of angiotensin-converting enzyme (ACE) was tested to verify our result. This study provides a valuable platform for gene expression and future functional research and serves as an example for similar studies in other insects.

## Materials and Methods

### Insect

*Ophraella communa* adults were collected from the fields in Laibin City in Guangxi Province in the summer of June 2017 and were maintained on common ragweed plants in the laboratory at the Chinese Academy of Agricultural Sciences (BJ, CAAS) for three generations. All the insects used for sampling were reared in an environmental chamber (PRX-450D, Ningbo Haishu Safe Experimental Equipment Co., Ltd., Zhejiang, China) at 27 ± 1°C, with 50 ± 10.0% relative humidity and a 14:10-hour photoperiod provided by artificial lights and with the robust common ragweed plants used as food.

### Experimental Sample in This Study

#### Samples of Developmental Stages and Sexes

The adults deposited their eggs in batches of about 35 on the underside of common ragweed leaves. The number of sampled individuals for each replicate across the different developmental stages and sexes of *O. communa* included 150 eggs, 30 first instar nymphs, 20 second instar nymphs, eight third instar nymphs, eight pupa (third pupa), five sexually immature (1 day old) male and female adults, and five sexually mature (8 days old) male and female adults. Three independent biological replicates were collected for each sample.

#### Tissue

Male adults (8 days old) of *O. communa* were dissected for samples of five different tissues, including head, testis, male accessory glands (MAG), fat body, and gut. Three independent biological replicates were collected with each group of 12 insects.

#### Male Reproductive System With a Different Body Size

The testis and accessory glands of large and small males, respectively, were dissected for these samples. Three independent biological replicates were collected with each group of 12 insects.

All the above samples were collected in 1.5-ml tubes, immediately frozen in liquid nitrogen, and stored at −80°C until use.

### RNA Extraction and cDNA Synthesis

Total RNA from all 48 samples was extracted using the Trizol reagent (Invitrogen, United States) according to the manufacturer’s protocol. The RNA integrity was tested by 1% agarose gel electrophoresis and quantified using NanoPhotometer^TM^ P330 (Implen, Munich, Germany), with A_260_/_*A*__280_ ratios ranging from 1.8 to 2.1 for all RNA samples. One microgram of total RNA was used to synthesize cDNAs using the TransScript One-Step gDNA Removal and cDNA Synthesis SuperMix (Transgen Biotech, Beijing, China) according to the manufacturer’s instructions.

### Reference Gene Selection and Primer Design

A total of 12 candidate reference genes that are most frequently used in qPCR investigations were selected from the *O. communa* transcriptome data (Table 1). The primers were designed for gene cloning and qPCR using the Primer five programs. All the candidate reference genes were PCR-amplified from the bettle’s cDNA using the corresponding primers (Table 1) and the pEASY-T3 Simple Cloning Kit (TransGen, Beijing, China). Six independent subclones were sequenced and accurate nucleotide sequences were obtained after the sequence analysis. All the verified reference gene sequences were submitted to the GenBank database.

**TABLE 1:** qPCR primers for candidate reference genes and characteristics of PCR amplification in *O. communa*.

Gene name	Accession number	Primer sequence (5′–3′)	Product length (bp)	Tm (°C)	E (%)	*R*^2^
*RPL4*	MN641110	F: TGTGGTAATGCTGTGGTAT	104	60	99.14	0.998
		R: TCTAGCACTGCATGAACA				
*RPS18*	MN641111	F: TCCGATGGCTGTATGTTAC	126	60	98.145	0.998
		R: GCTGTTGGGAGGAGTTAT				
*ACT1*	MN641112	F: CAGTGGTGATGGTGTTAC	148	60	97.263	0.999
		R: CTCAGGAGGTATGCCAAT				
*ACT2*	MN641113	F: GTTGCTCAGAGGTTATGC	149	60	105.63	0.997
		R: GGTAAGGTGTAAGGTTCCA				
*ARF1*	MN641114	F: GAGATGCCGTGTTGTTGA	99	60	102.166	0.997
		R: TGCGTAGCGAATGAAGAC				
*ARF4*	MN641115	F: GTGTAGATATGGAGCGAATG	98	60	103.345	1
		R: GTTAGCGAGCACTAGAATC				
*SDH*	MN641116	F: ACTTCCGCTCATACTTGT	149	60	98.306	0.999
		R: CCTCCTTCACCTCTACATC				
*GADPH*	MN641117	F: TGCTTCAACCGACACATT	136	60	102.673	0.999
		R: CGACCAATACGACCGAAT				
*EF1*α	MN641118	F: AGAACAATCCACCAAGAGG	98	60	101.81	0.996
		R: GTCCAACACAGGCGTATA				
*βTUBC*	MN641119	F: TCAGAGGTAGTCCTTCCA	103	60	100.58	0.996
		R: CAATTATCGCAGTTCTCCG				
*COX*	MN641121	F: GCTGAACACAGTTATTCTGA	150	60	98.533	0.998
		R: AGAGGCTCTATCTTGAAGT				
*RPL19*	MN641120	F: AAGGAAGGCATTGTGGAT	94	60	97.046	0.997
		R: GACGCAAATCTCGCATAC				

### Quantitative Real-Time PCR Analysis

Quantitative PCR reactions were performed using an ABI 7500 PCR Detection System (Applied Biosystems, United States). The qPCR reactions consisted of 10 μl of 2 × TransStart^TM^ Green qPCR SuperMix, 1 μl of the cDNA template, 0.4 μl of passive reference dye, and 0.4 μl each of the forward and reverse primers (10 μM) in a 20-μl final volume. The RT-qPCR procedure was as follows: an initial cycle at 94°C (30 s), followed by 40 cycles of 5 s at 94°C and 34 s at 60°C. Following the RT-qPCR procedure, the consistency and the specificity of the PCR-amplified products were subjected to melting curve analysis for all reactions. The amplification efficiencies (*E*) and correlation coefficients (*R*^2^) were determined for each gene using the standard curves with a 5-fold dilution series of the template (1/2, 1/10, 1/50, 1/250, and 1/1,250), where *R*^2^ is the slope of the standard curve. *E* was calculated according to the equation *E* = (10^[−1/slope]^ −1) × 100 (Michael and Pfaffl, 2001). Each reaction was performed in triplicate.

### Determination of Stability of Expression of the Candidate Reference Gene

The stability of the 12 candidate reference genes was evaluated using three different statistical programs, GeNorm (Vandesompele et al., 2002), NormFinder (Andersen et al., 2004), and BestKeeper (Pfaffl et al., 2004) under experimental conditions, and the instructions of all the three programs were consulted for the data arrangement and the subsequent analysis. Finally, the comprehensive ranking of each condition was obtained according to ReFinder. In addition, the number of reference genes for normalizing gene expression was decided by the pairwise variation (*V*_n/n__+__1_) which was performed using the GeNorm program. Universally, *V*_n/n__+__1_ less than the threshold value of 0.15 indicates that the most suitable number is *n*, and there is no need to introduce the n + 1 reference gene for normalization (Vandesompele et al., 2002).

### Validation of Reference Genes

Angiotensin-converting enzyme (*ACE*), a zinc-dependent peptidase (Isaac et al., 1999, 2007; Macours and Biol, 2004), was reported as a seminal fluid protein in *Drosophila melanogaster* and *Tribolium castaneum* (Rylett et al., 2007; Xu et al., 2013). The *ACE* gene (GenBank number: MN641122) cloning primers used were as follows: forward (5′-AATCCATGCCTACGTACGGT AT-3′) and reverse (5′-CTTCCCATCTCCACAGGTCA-3′). The normalization of *ACE* expression in different tissues was conducted using the most stable gene combinations (*COX*/*RPL19*) as determined by geNorm, the most single stable gene (*RPL19*), and the least stable gene (*SDH*)as determined by ReFinder. The relative quantification of the ACE gene was calculated using the 2^–ΔΔ*CT*^ method (Schmittgen and Livak, 2008). One-way ANOVA followed by Tukey’s least significant difference test was used to determine the significance of the *ACE* expression levels between different tissues (SAS 9.4).

## Results

### Acquisition of Candidate Reference Gene Sequences

The sequences of the 12 candidate gene, including *RPL4*, *RPS18*, *ACT1*, *ACT2*, *ARF1*, *ARF4*, *SDH*, *GAPDH*, *EF1*α, *βTUBC*, *COX*, and *RPL19*, were obtained from the transcriptome database of the testis and the accessory glands of *O. communa.* The sequences were verified by qPCR. All amplicons were sequenced and confirmed to exhibit 99–100% identity with the corresponding transcriptome sequence. The sequence information is listed in Supplementary Table S1.

### Specificity and Efficiency of RT-qPCR Primers

**FIGURE 1:**
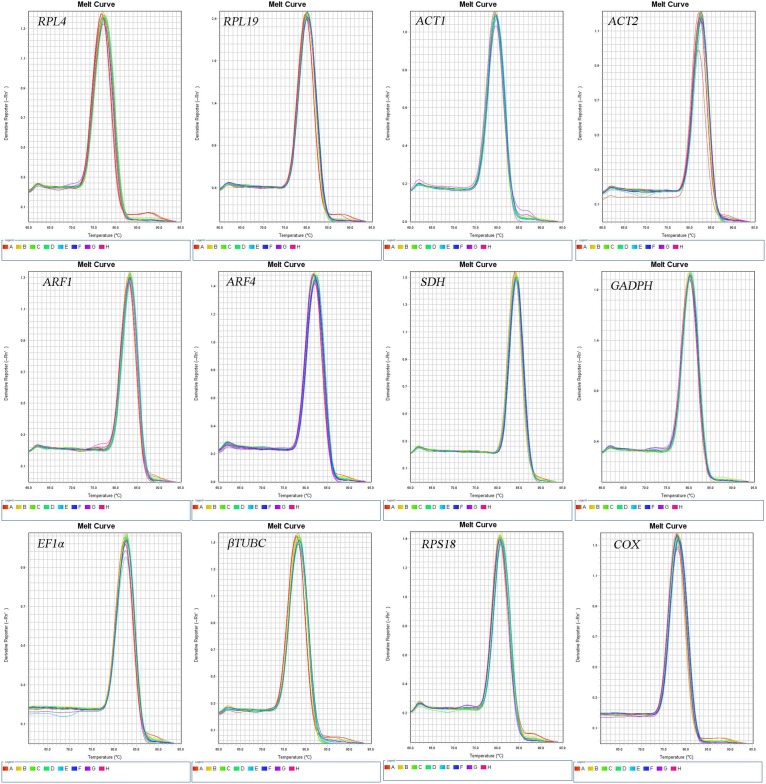
Melting curves of the 12 candidate reference genes in *O. communa*.

The accuracy of the RT-qPCR primers was confirmed by the presence of a single peak in the melting curve analyses and the absence of primer dimers, both of which also indicate the specificity of each primer pair (Figure 1). The amplification efficiency (*E*) of each primer (shown in Table 2) ranged from 97.05 to 105.63%, with associated *R*^2^ values of 0.996–1.000 (Table 1).

**TABLE 2:** Stability ranking of the 12 candidate reference genes’ expression in *O. communa* under different experimental conditions calculated by the three different analytical tools, geNorm, Normfinder, and BestKeeper, respectively.

Experimental conditions	Rank	Bestkeeper		NormFinder		geNorm	
		Gene	Stability	Gene	Stability	Gene	Stability
Developmental stage and sex	1	*RPL19*	0.179	*RPL19*	0.221	*RPL19/RPL4*	0.439
	2	*RPL4*	0.429	*RPL4*	0.347	–	–
	3	*ACT2*	0.514	*ACT1*	0.369	*RPS18*	0.525
	4	*ACT1*	0.559	*ACT2*	0.399	*ACT2*	0.604
	5	*EF1α*	0.653	*EF1α*	0.428	*COX*	0.775
	6	*SDH*	0.66	*SDH*	0.446	*ARF1*	0.818
	7	*RPS18*	0.66	*RPS18*	0.505	*ACT1*	0.865
	8	*COX*	0.759	*βTUBC*	0.589	*SDH*	0.913
	9	*ARF1*	0.793	*ARF1*	0.59	*ARF4*	0.971
	10	*ARF4*	0.885	*COX*	0.657	*GADPH*	1.029
	11	*βTUBC*	0.919	*ARF4*	0.695	*βTUBC*	1.095
	12	*GADPH*	1.15	*GADPH*	0.752	*EF1*a	1.206
Tissue	1	*RPL19*	0.24	*RPL4*	0.162	*COX/ARF1*	0.35
	2	*RPS18*	0.336	*COX*	0.294	–	–
	3	*COX*	0.361	*βTUBC*	0.301	*RPL19*	0.373
	4	*ARF1*	0.495	*RPS18*	0.354	*RPL4*	0.478
	5	*ACT2*	0.535	*RPL19*	0.385	*RPS18*	0.533
	6	*RPL4*	0.576	*ARF1*	0.406	*βTUBC*	0.614
	7	*βTUBC*	0.77	*EF1Aα*	0.491	*ACT2*	0.68
	8	*ARF4*	0.86	*ARF4*	0.562	*ARF4*	0.751
	9	*EF1α*	1.026	*ACT2*	0.656	*EF1α*	0.809
	10	*ACT1*	1.047	*GADPH*	0.773	*ACT1*	0.895
	11	*GADPH*	1.108	*ACT1*	0.79	*GADPH*	0.97
	12	*SDH*	1.23	*SDH*	0.843	*SDH*	1.038
Male reproductive systems with different body sizes	1	*RPL4*	0.065	*ARF4*	0.23	*RPL4/RPL19*	0.053
	2	*ACT2*	0.089	*ACT1*	0.271	–	–
	3	*RPL19*	0.105	*GADPH*	0.326	*ACT2*	0.169
	4	*GADPH*	0.397	*COX*	0.328	*ARF4*	0.245
	5	*RPS18*	0.552	*RPL19*	0.355	*GADPH*	0.369
	6	*EF1*a	0.736	*RPL4*	0.397	*ACT1*	0.516
	7	*ACT1*	0.736	*EF1*a	0.415	*EF1*a	0.584
	8	*COX*	0.809	*ARF1*	0.451	*COX*	0.661
	9	*ARF4*	0.846	*ACT2*	0.557	*ARF1*	0.708
	10	*ARF1*	0.97	*RPS18*	0.722	*RPS18*	0.788
	11	*βTUBC*	1.22	*SDH*	0.845	*SDH*	0.878
	12	*SDH*	1.297	*βTUBC*	0.88	*βTUBC*	0.969

### Expression Profiles of Candidate Reference Genes

**FIGURE 2:**
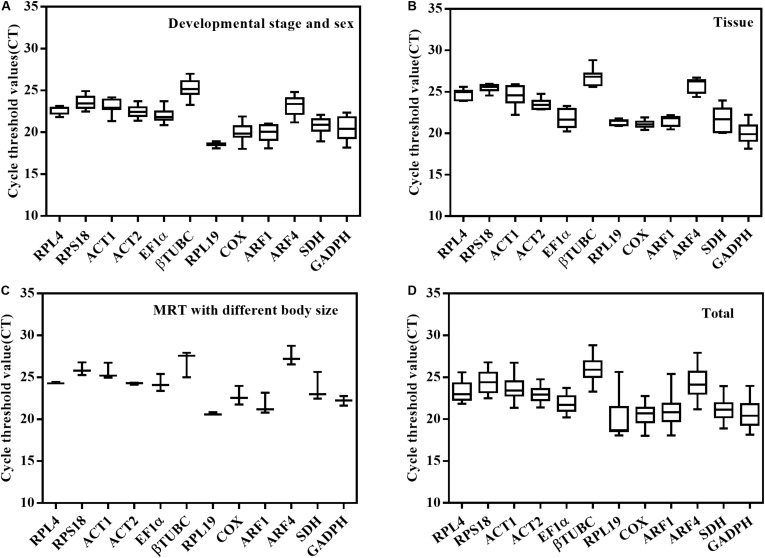
The expression profiles of the 12 candidate reference genes are shown as Ct values under three experimental conditions for *O. communa*. **(A)** Different developmental stages and sexes. **(B)** Different tissues. **(C)** Male reproductive systems with different body sizes. **(D)** Total Ct values exhibited different levels of transcript abundance in three biological samples.

The expression profiles of the 12 candidate genes were analyzed by qPCR, and the C_t_ values exhibited different levels of transcript abundance (Figure 2). For the different developmental stages and sexes, the mean C_t_ values of the 12 candidate reference genes ranged from 18.01 to 26.98 cycles. In different tissues, the C_t_ values ranged from 18.13 to 28.83. Even in males of different sizes, it varied from 20.58 to 28.76.

### Analysis of Stability of Candidate Reference Genes

**FIGURE 3:**
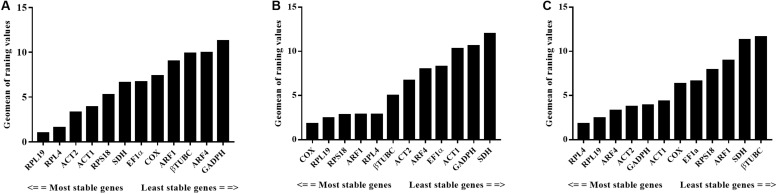
Expression stability ranking of the 12 candidate reference genes analyzed using ReFinder under three experimental conditions. **(A)** Different developmental stages and sexes. **(B)** Different tissue. **(C)** Male accessory glands from large and small males. The most stable genes are on the right and the least stable genes are on the left.

For different developmental stages and sexes—based on the analysis using GeNorm, NormFinder, and BestKeeper—the expression of *RPL19* varied the least, while *GAPDH* tended to fluctuate the most. According to ReFinder parameters, the comprehensive ranking in these samples was as follows: *RPL19* > *RPL4* > *ACT2* > *ACT1* > *RPS18* > *SDH* > *EF1*α> *COX* > *ARF1* > *βTUBC* > *ARF4* > GAPDH (Figure 3A). For the analysis of different tissue types, the stability of *RPL4* and *RPL19* were similarly excellent, while *SDH* and *GAPDH* were consistently recommended as the least stable by GeNorm, NormFinder, and BestKeeper. The comprehensive ranking was *COX* > *RPL19* > *RPL4* = *ARF1* = *RPS18* > *βTUBC* > *ACT2* > *ARF4* > *EF1*α> *ACT1* > *GAPDH* > *SDH* (Figure 3B). For the male reproductive system with different body sizes, the overall variability of the reference genes was low except for *βTUBC* and *SDH*. The overall ReFinder stability ranking (from most to least stable) was *RPL4* > *RPL19* > *ARF4* > *ACT2* > *GAPDH* > *ACT1* > *COX* > *EF1*α> *RPS18* > *ARF1* > *SDH* > *βTUBC* (Figure 3C). The stability of each sample’s stability was calculated using GeNorm, Normfinder, and BestKeeper, and all the specific values are shown in Table 2.

### Selection of the Optimal Number of Reference Genes to Normalize the Gene Expression

**FIGURE 4:**
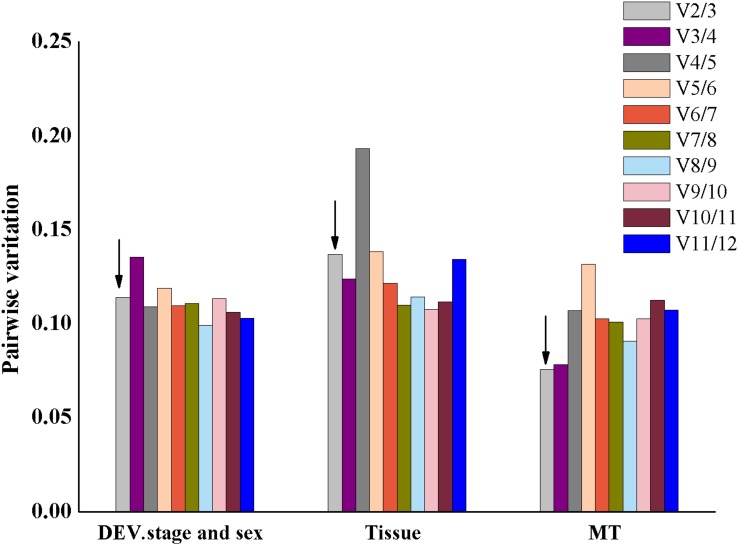
Pairwise variation in the three groups calculated by geNorm is required for accurate normalization of gene expression. The threshold used was 0.15. The arrow indicates that the value of V_2_/V_3_ is less than 0.15.

We used GeNorm to compute the variation value to ensure the optimal number of reference genes. As shown in Figure 4, the V2/3 was below 0.15 across the different experimental conditions, which indicated that two reference genes were reliable for an accurate normalization of gene expression.

### Validation of Selected Reference Genes in *O. communa*

**FIGURE 5:**
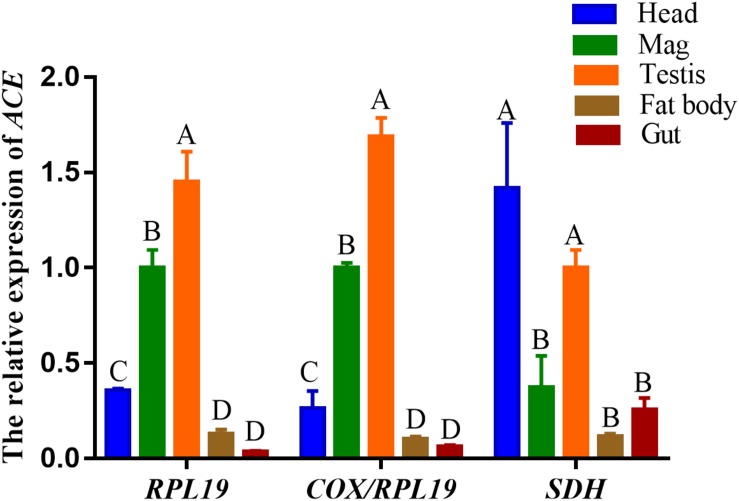
Relative quantification of *ACE* genes in different tissues. The expression levels of *ACE* genes were normalized using the best reference paired *COX*/*RPL19*, the single best stable gene *RPL19*, and the reference gene SDH that changed the most in tissues. Values are means ± SE. Different letters indicate significant differences in gene expression among the different tissues of *O. communa* (*P* < 0.05).

The *ACE* expression level was used to verify the validity of the reference genes between the different tissues. The best paired, best single, and the worst single reference genes—*COX*/*RPL19*, *RPL19*, and *SDH*, respectively, were used as internal controls. The *ACE* expression patterns were similar in that the highest expression of *ACE* was in the male reproductive tissues (testis and MAG) when normalized by the paired *COX*/*RPL19* and single *RPL19* (Figure 5). However, when we used *SDH* to normalize the expression levels, the highest expression level of *ACE* was in the heads and significantly higher than in the other tissues (Figure 5). This conclusion was completely inconsistent with the results seen through the normalization with *RPL19* or *COX*/*RPL19*. This indicated that the accuracy of the RT-qPCR results may be influenced by the instability of the reference gene, which might even lead to an inaccurate or wrong conclusion.

## Discussion

*Ophraella communa* is a very valuable and effective biological control agent of *Ambrosia artemisiifolia* (Guo et al., 2011). Unfortunately, research on the molecular mechanisms of *O. communa* is scarce, making the study of its physiological and biochemical behavior difficult. Recently, the male transcriptome of *O. communa* was sequenced using Illumina high-throughput sequencing technologies (unpublished data), and further research on functional genomics and related gene expression studies will be initiated soon.

The quantitative accuracy of gene expression relies on the reference gene (Lu et al., 2018b). In our study, based on the study of reference genes in Coleoptera, 12 candidate reference genes, including traditional genes and novel genes, were selected. These genes have been evaluated as the most stable genes in different insects: *RP18*, *RP4*, *AFR1*, and *ARF4* in *Leptinotarsa decemlineata* (Shi et al., 2013), β*-actin* and *EF1a* in *Diabrotica virgifera virgifera* (Rodrigues et al., 2014), *Actin* and *RPS18* in *T. castaneum* (Lord et al., 2010; Toutges et al., 2010; Sang et al., 2015), *EF1*α, *ACT1*, and *RPL19* in *Colaphellus bowringi* (Tan et al., 2015), *SDHA*, *ACT* and *TUB* in *Galeruca daurica* (Tan et al., 2017), *GADPH* in *Hippodamia convergensin* (Pan et al., 2015), and *COX* in *Agasicles hygrophila*. Besides that, these candidate genes have also been recommended for normalization gene expression in others (Lu et al., 2018b; Shakeel et al., 2018). Three different methods, GeNorm (Vandesompele et al., 2002), NormFinder (Andersen et al., 2004), and BestKeeper (Pfaffl et al., 2004), were used to identify the stability of the 12 candidate genes across the condition of developmental stages and sexes, different tissues, and male reproductive system with different body sizes. However, because each method has its own strengths and appropriate application conditions, the expression stability obtained by different algorithms was different (Table 2). Therefore, another analysis tool, ReFinder (Yang et al., 2015a), was used to integrate the variation in the results between them and to define an overall final ranking (Xie et al., 2012).

Our current research focuses on the molecular mechanisms of the male fitness differences relative to different body sizes. The results of this study displayed that the reference gene transcript levels vary with the above experimental conditions (Figure 2). For the sample of different developmental stages and sexes, contrary to the results of other studies in Coleoptera insects, GAPDH, *EF1*α, and *TUB* performed the best stability under this condition (Rodrigues et al., 2014; Pan et al., 2015; Yang et al., 2018). Our results demonstrated that *RPL19* and *RPL4* showed the most stable gene expression, while *GAPDH*, *EF1*α, and *βTUBC* showed the least stability (Figure 3A). All four algorithms obtained the same two top-ranked genes with few differences in their respective orders. Similar to our results, in *L. decemlineata*, *RPL4* and *RPS18* exhibited excellent stability in different developmental stages and sexes. Ribosomal protein (RP) also presented superior stability in *Coccinella septempunctata* across this condition (Shi et al., 2013; Tan et al., 2015; Yang et al., 2016). For different tissues—consistent with the results in *A. hygrophila*—a novel reference gene, *COX*, that encodes for a key enzyme (Liu et al., 2017) and that responds to a variety of metabolic states showed the least variation (Figure 3B). In addition, like the ranking for developmental stages and sexes, the RPs, including *RPL19*, *RPLl4*, and *RPS18*, displayed high stability (Figure 3B), while *SDH* and *GADPH* were identified as the least stable genes. For the male reproductive system with different body sizes, the variation of each candidate gene is relatively small compared with the first two conditions (Figure 2C), and a similar ranking trend with previous condition was observed in this sample. *RPL19* and *RPL4* performed the least variation. To our knowledge, studies on the reference gene for body size are scarce. Our results provide a reference for the gene expression in other insects with different body sizes. Integrating the excellent stable reference genes under three conditions, *RPL19* can be used to normalize the gene expression. Studies in *C. bowringi* similarly concluded that the *RPL19* presented excellent stability across developmental stage, sex, and tissue (Tan et al., 2015). In *D. virgifera virgifera*, *EF1a* and *Actin* were respectively identified as the most stable gene among different tissues and developmental stages (Rodrigues et al., 2014). However, our results were almost the opposite. Therefore, the choice of the control gene for *O. communa* is of utmost importance.

Coincidentally, our results verified that RP gene expression was extremely stable under each experimental condition. Generally, ribosomal protein genes are expressed in all types of cells for the synthesis of new ribosomes, and RPs are among the most highly conserved proteins across all life forms (Hsiao et al., 2001). Recently, researchers found that even in *T. castaneum*, most *RP* gene expression was maintained stably following a fungal challenge (Lord et al., 2010; Lu et al., 2018b). In addition, besides Coleoptera insects, ribosomal genes showed high stability in diverse abiotic and biotic conditions in other organisms (Jonge et al., 2007; Popovici et al., 2009; Tong et al., 2009; Zhong et al., 2013; Yang et al., 2015b, 2016; Ma et al., 2016; Yan et al., 2016; Lu et al., 2018a; Luo et al., 2018). Our screening results also demonstrated that the *RP* gene is transcriptionally conserved under different physiological conditions. However, under some conditions, the expression levels of RP-encoding genes may be unstable. For example, *RPS3* was the least stable gene in *Spodoptera litura* under different populations, exposed to different temperatures, or treated by different insecticides (Lu et al., 2013). Moreover, other classic references are *GADPH*, *TUB*, and *Actin* which have poor stability in *O. communa* (Figure 3). Therefore, it is very unwise to choose a reference gene blindly regardless of the conditions and species.

Furthermore, to certify the reliability of the reference gene in *O. communa*, the relative transcriptional level of the *ACE* gene was determined in different tissues using qPCR. GeNorm recommended that the use of two reference genes was optimal for normalization under each experimental condition (Figure 4). Therefore, *ACE* expression was normalized by the single stable *RPL19*, the pairwise stable *RPL19* and *COX*, and the least stable *SDH* alone. Consistent with a previous study (Xu et al., 2013), when *RPL19*/*COX* and *RPL19* alone were used as internal controls, *ACE* expression was found to be abundant in the reproductive organs, including the testis and the MAG. In contrast, the head and the testis showed a high *ACE* expression when normalized using *SDH* (Figure 5). This implies that different controls may lead to different conclusions. Therefore, we strongly recommend the validation of the stability of the reference gene expression prior to performing RT-qPCR experiments. Our study found that *RPL19* and *RPL4* showed excellent stability among the candidate reference genes for all three experimental conditions. This provides a fundamental premise to analyze qPCR data in *O. communa* and to further clarify the physiological and biochemical mechanisms in this important natural ragweed enemy.

## Conclusion

In summary, we identified several stable housekeeping genes that are suitable as reference genes in *O. communa*. This is the first study to report the reference genes and to establish a standardized RT-qPCR analysis protocol in *O. communa*, which will contribute to the development of molecular research on this vital insect.

## Data Availability Statement

The datasets generated for this study can be found in the NCBI using accession MN641110, MN641111, MN641112, MN641113, MN641114, MN641115, MN641116, MN641117, MN641118, MN641119, MN641121, MN641120, and MN641122.

## Ethics Statement

The animal study was reviewed and approved by Ethics of Animal Experiment of Institute of Plant Protection, Chinese Academy of Agricultural Sciences.

## Author Contributions

ZZ designed the study and revised the manuscript. YZ performed the research and wrote the manuscript. JC applied software for data analysis. GC searched for the sequence of candidate reference genes. CM, XG, ZQT, SC, and ZYT participated in the sample collection and data sorting. HC, JG, and FW guided in the data analysis.

## Conflict of Interest

The authors declare that the research was conducted in the absence of any commercial or financial relationships that could be construed as a potential conflict of interest.
