# Supplementary material for: Identification and Validation of Reference Genes for Quantitative Gene Expression Analysis in Ophraella communa
Source: Front Physiol. 2020 May 7;11:355. doi: 10.3389/fphys.2020.00355 (PMC7220992; doi:10.3389/fphys.2020.00355)
Supplement: Supplementary file 1 [file Table_1.DOCX]

Supplementary table 1 All reference gene amplification primers in this study

| Gene name | Accession Number. | Primer sequence (5’-3’) | Product length(bp) |
| --- | --- | --- | --- |
| *RPL4* | MN641110 | F:AACAGGTGACTCCACAGAACA  R: AACTCCACCGCCCTTAAACA | 833 |
| *RPS18* | MN641111 | F:TGTATAGGGCATAAGGTTGT  R: TTCTTGCTGTTGGGAGGAGT | 450 |
| *ACT1* | MN641112 | F: AGCAGTTCAAGCAGTATTAGCCT  R:AAGCCAACATACTTCCACCAAACCA | 734 |
| *ACT2* | MN641113 | F: ACCTCCCATGAACCCGACTA  R: TCATCACTTCCGCCAACACA | 764 |
| *ARF1* | MN641114 | F: CCAATGCGACACGCTGATTC  R: TGCGTAGCGAATGAAGACCC | 614 |
| *ARF4* | MN641115 | F: TCTTGCGTAGTTGCCCCAAG  R: CGGTAATAGCGCAAGCTGGT | 569 |
| *SDH* | MN641116 | F:TTTCCTGCACTTCCGCTCAT  R:AACCGGTCGGTAATCGATGG | 1142 |
| *GAPDH* | MN641117 | F: TGCTTCAACCGACACATTTA  R: ATGACAGGGTCGTCTGGGTT | 526 |
| *EF1α* | MN641118 | F: AGGCACAAGAGATGGGCAAA  R: GACAGTCCAACACAGGCGTA | 957 |
| *βTUBC* | MN641119 | F: AAAGTGTCCCAGTGAATGAGTT  R: ACATATCCAGGGGTGCCAAG | 916 |
| *Cox* | MN641121 | F:CGATAGGGGCAGTATTTGCT  R:ATCATTGGTGGCCGATTGTT | 777 |
| *RPL19* | MN641120 | F: GGAAGGCATTGTGGATTTGG  R: CATTACCCTTAGCTTTCATG | 175 |
